# Jesters of Well-Being: Examining the Relationship between Clown Doctors and Patients

**DOI:** 10.3390/bs14050398

**Published:** 2024-05-10

**Authors:** Alberto Dionigi, Alessandra Fermani, Carla Canestrari

**Affiliations:** 1 Noesis Clinical Center, 47841 Cattolica, Italy; albe.dionigi@gmail.com; 2 Department of Education, Cultural Heritage and Tourism, University of Macerata, 62100 Macerata, Italy; alessandra.fermani@unimc.it

**Keywords:** clown doctors, humor, comic styles, healthcare clowning

## Abstract

Clown doctors play a crucial role in enhancing the well-being of patients through the use of humor. However, little is known about how the use of humor by clown doctors changes in relation to the developmental age of patients. This research explores the interplay between the type of humor used by clown doctors, their experience (in terms of years of clowning and type of clowning), and the developmental age of the patients (children, adolescents, adults, elderly). Data for this cross-sectional study were collected through an online survey distributed to 210 Italian clown doctors (143 females, 67 males), aged between 18 and 75 years (M = 47.34, SD = 12.31), affiliated with different Clown Care Units. The survey included the Comic Styles Markers, questions on the patients’ developmental age, type of clowning (Auguste vs. Whiteface), and years of experience. The findings enhance our understanding on how clown doctors interact with patients of different developmental ages. The discussion draws connections to previous studies conducted on groups of clown doctors, providing a broader context for understanding the implications of humorous interactions in this unique healthcare domain.

## 1. Conceptualization of Hospital Clowns

Clowning in healthcare settings is a well-established practice of providing entertainment for individuals across various developmental age groups during their recovery process [1]. Over the past 20 years, a substantial amount of scientific research has assessed the impact and effectiveness of clowns in medical environments; however, few studies have assessed the artistic traits of this character, particularly focusing on the type and utilization of humor. Therefore, this study aimed to explore the connections between clown doctors’ regular use of both harmless and harmful humor and the developmental ages of their patients. According to previous research, the clown is a unique and intriguing character that projects himself/herself into a fictional world, at a wavelength that captivates attention and defies convention [2]. As an artist, the clown adopts a playful and childlike demeanor, focusing on evoking positive emotions and establishing a close connection with the physicality of the performer. However, in contrast with traditional actors, clowns do not merely play a predetermined role but develop their unique characters, drawing inspiration from both the physical and psychological nuances of their settings and their performance [3]. This approach lends a unique and personalized touch to each clown’s persona.

Clowns are generally categorized into two traditional types distinguished by unique makeup styles and personalities: the Whiteface and the Auguste. The Whiteface and the Auguste clowns represent contrasting characters. The Whiteface is elegant, serious, and authoritative, representing structure, order, and preparation; in contrast, the Auguste is clumsy, silly, and playful, symbolizing spontaneity, disruption, and impulsiveness. Typically, there is a conflict between them.

The activities of professional clowns as members of hospital healthcare teams commenced in 1986, when Michael Christensen, a professional clown working at the Big Apple Circus in New York, founded the Big Apple Circus Clown Care [4]. Over the following years, the main aim of clown doctors was to bring smiles, laughter, and entertainment to patients and, thus, make their hospitalization less traumatic. This led to the successful establishment of numerous clown care units (CCUs) worldwide over the past three decades. Simultaneously, similar units emerged across the world, extending their focus beyond children to include adolescents, adults, the elderly, and even the medical staff itself, considering the concomitant positive healthcare outcomes [5]. Whereas in the beginning, clown practice was mainly designed for children, over time, clown doctors expanded their practice to include people of different developmental ages; nowadays, adolescents, adults, and elderly are the typical recipients of their intervention.

### 1.1. Review of Research on the Role and Effectiveness of Hospital Clowns

To date, scholars have commonly claimed that the presence of a clown in healthcare settings can have a positive impact on children, adults, and the elderly; however, most of the related studies have been conducted on young patients, with only a few of them regarding older recipients [6,7]. In general, the relevant studies have focused on both analyzing positive experiences (in terms of their contribution to the well-being of individuals) and examining the reduction in negative experiences, e.g., the alleviation of pain and other effects associated with illness [6,8,9]. Moreover, although studies regarding the effectiveness of healthcare clowns have been widely tested, only a few investigations have evaluated the artistic characteristics of this character, especially with respect to the kind and use of humor by clowns. Although the clown is a comic character that primarily utilizes humor in their interactions, and research has shown how humor-based interventions by clown doctors can mitigate the negative effects of hospital visits for children, humor has been deemed the subject of only a limited number of studies concerning the artistic characteristics of clown doctors. The few related studies that have explored this aspect have generally confirmed the crucial role of adaptive humor used by clowns. For example, a qualitative study conducted in Israel was aimed at gaining a better understanding of the emotional issues experienced by clown doctors in their work with adults affected by chronic diseases [10]. Here, humor emerged as a fundamental skill for clowns, serving as a tool that enabled patients to address challenging situations in light of its important role as a stress management and coping strategy.

Linge [11] conducted a meta-analysis concerning a seven-year research project that was aimed at achieving a more sophisticated and deeper psychological understanding of the unique encounters between hospital clowns and hospitalized children. Qualitatively evaluating the relationship between the clowns and the recipients of their intervention, Linge found that positive humor enhances hospital clowns’ ability to empathetically engage with their patients. Indeed, in this relational frame, humor helps children to find relief and to see the brighter side of their conditions.

Moreover, a recent qualitative study involving therapeutic clowns in Canada emphasized that both playfulness and creativity are essential skills required for effective in-person or online therapeutic work [12]. Additionally, a study conducted on Israeli Medical Clowns that triangulated 26 video-recorded simulations and conducted 12 in-depth semi-structured interviews with clowns identified 40 distinct therapeutic skills of such clowns. Here, humor emerged as a very important skill even though it was one among many others. Moreover, this study outlined five primary therapeutic goals for medical clowns: fostering relationships, addressing emotions, encouraging a sense of control, providing care and encouragement, and promoting adherence to treatment [13].

Although it is generally evaluated positively, one must underline that humor is not always purely positive; it can have negative connotations or undertones depending on the therapeutic context and the individuals involved [14]. Numerous studies have suggested that humor can also have a problematic and unhealthy aspect [15]. This perspective highlights the need for a more fine-grained understanding of humor that acknowledges its potential for both positive and negative therapeutic effects. Moreover, it would be helpful to determine whether various negative forms of humor might characterize different clown characters (e.g., the Whiteface clown) as part of a comic style.

### 1.2. The Comic Style Markers Approach 

In this context, a recent development known as Comic Style Markers (CSM) has been introduced [14] with the aim of enhancing the effectiveness of in-depth investigations into the nature of humor. This approach focuses on eight lower-level comic styles that can be categorized as either lighter or more complex/problematic expressions of humor, collectively encompassing fun, humor, nonsense, wit, irony, satire, sarcasm, and cynicism [14]. The four lighter styles, associated with benign and social affect, behaviors, cognitions, and goals, are the following: (a) fun: aimed at spreading a positive mood and fostering good companionship; (b) humor: aimed at evoking sympathy towards human shortcomings, identifying discrepancies in everyday experiences, and treating such discrepancies in a humorous and benevolent manner; (c) nonsense: entails experimenting with incongruities and ridiculousness without a specific purpose; (d) wit: involves the ability to establish clever connections between ideas and thoughts. On the other hand, the more complex/problematic styles, which lack this benevolent affect, are primarily centered around mockery and ridicule: (a) irony: reflecting a contrast or incongruity between expectations regarding a situation and its reality, characterized by the expression of the opposite of the intended meaning; (b) satire: directed at criticizing and correcting shortcomings, misconduct, and moral wrongdoings with the intention of improving the world; (c) sarcasm: grounded in the need to be critical of others and convey contempt; (d) cynicism: aimed at devaluing commonly recognized values.

To date, the only empirical study assessing whether clown doctors possess specific differences in terms of humor, compared to laypeople as well as related to their character, has been conducted in Italy [16]. This study has asserted the following. Compared to the larger populace, these clown doctors possess higher levels of fun, benevolent humor, and nonsense, along with a lower level of cynicism; moreover, individuals with greater concomitant experience generally exhibit a reduced usage of irony, sarcasm, and cynicism compared to those with less experience; further, playfulness is predominantly associated with lighter forms of humor, and there are distinct variances in this regard between the Whiteface and Auguste clown doctors.

A recent study was undertaken to investigate the fear of being ridiculed (gelotophobia) and humor coping mechanisms among a group of individuals comprising hospital clowns, people who attended healthcare-relevant training courses related to humor, and people who attended healthcare or professional training courses not related to humor (controls) at an Italian children’s hospital [17]. Results revealed that hospital clowns exhibited the lowest fear of being laughed at, followed by individuals undergoing humor training and controls. This aligns with expectations, as hospital clowns and those training to incorporate humor into their healthcare roles actively seek out and create situations to induce laughter in patients. Conversely, participants with tendencies toward gelotophobia were anticipated to avoid situations involving laughter.

Owing to the shortage of studies on the role of specific dimensions of clown doctors’ humor in their interactions with patients of different developmental ages, the authors of the present study decided to evaluate this aspect by conducting research on a large sample of clown doctors. Moreover, considering that the majority of related studies in this field have not considered the differences between novel and experienced clowns, the present study also considered the extent of experience of these practitioners, i.e., the number of years spent practicing, in order to investigate the relationship between the humor used and its diverse recipients in a better way. 

### 1.3. Aim of the Study

This study attempted to investigate the relationships between the habitual use of benign and malicious humor by clown doctors, their concomitant experiences, and the developmental ages of their patients. The study directly addressed these issues by examining how specific categories of humor styles related to the clown doctors’ years of experience and type/s of clowning. Additionally, it delved into the relevant interplay between different recipient groups, including children, adolescents, adults, and the elderly.

## 2. Materials and Methods

### 2.1. Participants 

The sample of this research comprised 210 clown doctors (143 females, 68.1% of the sample; 67 males, 31.9% of the sample) aged between 18 and 75 years (M = 47.34; SD = 12.31). All participants were well-educated adults (0.5% with primary schooling; 8.6% with low secondary schooling; 52.4% with upper secondary schooling; 28.1% with university education; 7.6% with master’s/postgraduate degrees; and 2.9% with doctorates). Regarding marital status, 30.5% of the participants were unmarried, 57.6% were married or were cohabiting, 9.5% were divorced, and 2.4% were widowed. Moreover, the participants had varying levels of experience in the art of clowning in healthcare settings (M = 7.16 years; SD = 5.32; range = 0–22 years). Based on their years of experience, we separated the clown doctors into three subgroups (eight were missing): “<1–4 years” (74; 36.6% of the sample); “4–9 years” (63; 31.2% of the sample); and “>9 years” (65; 32.2% of the sample). Notably, the majority of the participants (N = 195; 92.9%) were volunteer clowns. Furthermore, 122 (58.1%) of them commonly played the role of the Auguste clown, while 88 (41.9%) commonly played the role of the Whiteface clown.

### 2.2. Instruments 

The participants provided anonymous responses to a brief demographic questionnaire, wherein they disclosed details such as their age, gender, level of education, and marital status. This questionnaire also included inquiries about the clown doctors’ engagement; the participants were required to specify the primary character they portrayed (Whiteface or Auguste), their experience (in terms of years and type of clowning), and the developmental age of their patients (children, adolescents, adults, elderly, etc.).

Importantly, the CSM [14] used in this study comprised forty-eight items, involving eight subscales (six items per style), each of which reflected a distinct comic style: Fun (e.g., “I am a funny joker”), Benevolent Humor (e.g., “When my humor is aimed at human weaknesses, I include both myself and others”), Nonsense (e.g., “I like nonsensical humor”), Wit (e.g., “I have the ability to tell something witty and to the point”), Irony (e.g., “Whoever understands my irony is, along with me”), Satire (e.g., “I parody people’s bad habits to fight bad and foolish behavior”), Sarcasm (e.g., “Biting mockery suits me”), and Cynicism (e.g., “I tend to show no reverence for certain moral concepts and ideals, but only scorn and derision”). All items were scored using a seven-point Likert scale ranging from 1 (strongly disagree) to 7 (strongly agree). The total scores corresponded to the mean of the six abovementioned items, with higher scores corresponding to the higher use of a specific comic style. Notably, this study used the Italian version of the CSM [18]. Importantly, the eight scales utilized by this study showed good-to-acceptable reliabilities (McDonald’s ω total: Fun = 0.84; Humor = 0.79; Nonsense = 0.83; Wit = 0.82; Irony = 0.76; Satire = 0.78; Sarcasm = 0.73; Cynicism = 0.82).

### 2.3. Procedure 

This study had a cross-sectional research design, involving online data collection. Its inclusion criteria encompassed individuals aged 18 years or older and holding Italian citizenship. Thus, emails were sent to various CCUs inviting affiliated clowns to undergo a battery of tests. These emails included a link to a survey hosted on Survio.com, which ensured participants’ anonymity. A total of 253 individuals responded to the survey, with the final sample comprising 210 clowns who fully completed the battery of tests. More precisely, the survey included an explanation of the study’s purpose and a form requesting the participants’ agreement. Moreover, it adhered to the local ethical guidelines, thus gaining approval from the ethical research committee of the University of Macerata.

### 2.4. Data Analysis

In this study, the descriptive statistics, multivariate analysis of variance (MANOVA), bivariate correlations, and linear regressions of the study data were computed. All statistical analyses were performed using the SPSS v.21.0 statistic software package (IBM Corp., Armonk, NY, USA). All values of *p* < 0.05 were considered to be statistically significant.

## 3. Results

### 3.1. Multivariate Analysis of Variance (MANOVA)

First, the present study examined whether the participants differed in terms of individual levels of the eight CSM (Fun, Humor, Nonsense, Wit, Irony, Satire, Sarcasm, and Cynicism); the target groups of the intervention (children, adolescents, adults, and elderly) were deemed dependent variables, while the three levels of experience (i.e., <1–4 years; 4–9 years; >9 years) and the two characters (Auguste clown and Whiteface clown) were considered as independent variables. These decisions were made according to the following aims of the study: to describe and test the impact of the clown doctors’ characters and experience on their comic styles and the developmental ages of their patients. Notably, the analyses by this study did not reveal any statistically significant differences with respect to the clown doctors’ gender and age.

In turn, this study found that the participants (see Table 1) reportedly carried out their activities predominantly with children and less so with the elderly. With respect to each intervention group (children, adolescents, adults, and the elderly), significant differences were found in the MANOVA only regarding the activities involving adults. In addition, those with less experience reportedly worked less with adults than those with more (years of) experience.

As markers, Humor and Nonsense collected very high scores from all the study groups (categorized in terms of the three abovementioned levels of experience and the two characters); however, no statistical significance was found in this regard. Instead, the results of the MANOVA analyses revealed significant statistical differences for Irony, Sarcasm, and Cynicism in light of Tukey’s test, which was conducted for all the study groups (two characters and three levels of experience).

Moreover, the participants showed high-level scores regarding all adaptive styles (higher scores than the mid-level score of the Likert scale: 4) and low scores in two out of the four problematic styles (i.e., Sarcasm and Cynicism). Specifically, those with higher experience (>9 years) were found to employ less irony, sarcasm, and cynicism than those with less experience (<9 years), as evidenced by Tukey’s test.

Regarding the clown doctors’ roles, according to Wilks’ Lambda criterion, the Whiteface clown doctors reportedly used more sarcasm and cynicism than their Auguste counterparts. These results indicated that in combination, the abovementioned dependent variables were significantly affected by the following factors: Sarcasm (F [1, 210] = 4.34, *p* < 0.039, η^2^ = 0.02) and Cynicism (F [1, 210] = 4.48, *p* < 0.036, η^2^ = 0.02); however, no interaction was observed between experience and role. In addition, no significant interactions were evident even with respect to the developmental age of patients and the clown doctors’ role.

### 3.2. Correlations and Regressions

To further examine the relationship between group intervention, levels of experience, and the eight comic styles, the present study performed statistical correlations and linear regression analyses concerning the entire study sample (see Table 2). 

In turn, the correlations in the entire sample revealed that only the clown doctors’ level of experience and Irony showed significant differences. Experience was positively associated with adolescents, adults, and the elderly; the more experience clown doctors had, the more they tended to work with adolescents, adults, and the elderly. Meanwhile, Irony was negatively associated with children and adolescents; clown doctors working with children and adolescents tended to use less irony than those working with adults or the elderly. 

The linear regression findings reported in Table 2 indicated a moderate R squared effect. Nevertheless, in general, the results of this study were noteworthy. In the entire sample, according to the regression analyses, the following were found; experience was positively associated with the children, adolescents, adults, and elderly, whereas Irony was negatively associated with children and adolescents. No other significant results were found.

## 4. Discussion

In this study, we cross-sectionally investigated how specific categories of humor used by clown doctors, explicitly the comic styles, were related to their role, their professional experiences, and the developmental ages of the patients with whom they tended to interact (children, adolescents, adults, and the elderly). Overall, the study’s findings supported our hypotheses that clown doctors’ use of humor is associated with differences in their role, experience, and the developmental age of their patients. It should be noted that the magnitude of the effect sizes is statistically significant, but of small effect size. In any case, the results are noteworthy.

Specifically, the results showed that the participants carried out their activities predominantly with children and less often with the elderly. This result aligned with the following findings of the existing literature; clowning in healthcare settings was first established with the aim of alleviating the distress, discomfort, and anxiety experienced by children enduring chronic illnesses and prolonged hospitalization [4]. Over recent years, the work of clown doctors was integrated in a large variety of settings with patients of different developmental ages; nowadays, however, the majority of the interventions by clown doctors are directed towards pediatric patients [5]. Nevertheless, clowns are putatively associated with fun and humor, as they wear colorful costumes and behave in a funny and incongruent manner that provokes children’s laughter [19]. The essence of clowning lies in embracing one’s flaws and weaknesses, which turns clowns into sources of comedy in light of their constant and direct engagement with their audience [20]. Hence, they may be less appreciated by adults as compared to children; indeed, older adults may change in terms of their ability to be cognitively flexible [21] and may come across as less playful [22]. 

In this regard, this study’s findings revealed that clown doctors with less experience work less with adults than those with more years of experience. Specifically, the more experienced clown doctors are, the more they tend to work with adolescents, adults, and the elderly. An inexperienced person approaching clowning activity must undergo a specific training program adumbrated by the CCU of which they are a part. The same CCU may engage in active collaborations and agreements with specific hospital departments (e.g., pediatrics, oncology, adult care departments); the clown doctor must make a decision regarding the specific department with which he/she wants to engage. One can assume that less experienced clowns may prefer to interact mainly with children because these are the recipients who enjoy clown activity the most and are comparatively more inclined to laugh and play with clown doctors. Therefore, people approaching clowning in the domain of healthcare may prefer to start with an easier audience and subsequently, as they gain more experience, they can incorporate more complex audiences and situations, e.g., approaching less eager recipients such as adults. In other words, the activity of clown doctors is related to the CCU to which they belong, and the initial training performed by future clown doctors is given by the same CCU in accordance with each clown doctor’s mission. 

Regarding the different kinds of humor, this study’s results showed that with respect to the positive and benevolent role of clown doctors in healthcare settings, the participants showed high-level scores for all adaptive styles and low-level scores for Sarcasm and Cynicism. Considering that the main aim of clowning is to induce positive emotions, relieve stress, and mitigate negative emotions [8], we therefore assumed that clown doctors use gentle play, distinguished by adaptive forms of humor [23]. This finding aligned with those regarding the salient role of clowns in healthcare environments: providing affiliative and positive forms of humor to entertain their audience and to inhibit the use of maladaptive humor that could harm their patients [24]. In terms of the roles assumed, the Whiteface clown doctors exhibited a higher tendency to use sarcasm and cynicism compared to their Auguste counterparts. Moreover, the Whiteface clown reportedly represented the rational voice of reason and served as a disciplined decision-maker characterized by strictness, authority, severity, and precision. These attributes have been previously associated with both sarcasm and cynicism [25]. In terms of the interaction of the two clown characters, the Whiteface clowns tended to use sarcasm and cynicism to make fun of their colleague embodying the role of the Auguste clown, creating a comic conflict that would end in a shared amusement on part of the audience [20].

Further, this study asserted that the more experienced clowns (>9 years) were found to employ less irony, sarcasm, and cynicism than those with less experience. A possible explanation for this finding could be the following; novice clowns, initially approaching their activity with only a basic level of training, tend to use categories of humor that can generally be more offensive and may be linked to their own way of expressing humor. Over time, concurrently gaining more experience, clown doctors gradually learn to regulate their use of humor and employ more adaptive forms of the same, focusing their attention on modes of playfulness that are more suitable.

Finally, this study’s results highlighted that clown doctors who worked with children and adolescents used less irony than those working with adults or the elderly. This result also aligned with those of the existing literature; studies on developmental humor have shown that children are less accurate in comprehending ironic meanings compared to literal meanings (e.g., [26,27,28,29,30]). Humor is primarily a cognitive process that involves various cognitive mechanisms such as incongruity detection, pattern recognition, and surprise [31]. On the other hand, irony is a comic device that necessitates utterances that are different from or even opposite to what they mean. Understanding irony requires cognitive flexibility and the ability to recognize and interpret what is really meant by the speaker. This process involves advanced cognitive reasoning skills that allow a person to grasp the intended interpretation behind an ironic expression (e.g., [32,33]). Thus, it is not surprising to find that clown doctors seldom use irony when engaging with groups of children. In fact, a substantial body of research indicates that the ability to recognize irony typically develops in children as they approach six years of age [28,30,34]. Interpreting irony is, therefore, difficult for children and this ability improves with age [32]. Considering the limited understanding of verbal irony by children of a young age, clowns use it sparingly with child patients and, in this respect, focus instead on different styles of humor, along with approaches such as physical forms of entertainment. 

Overall, the findings of this study reveal clown doctors’ specific utilization of different kinds of humor depending on the type of patients with whom they are required to interact. Although these results are promising, some limitations of the study need to be acknowledged. First, the correlational nature of the study did not permit the detection of causality. Although we received useful indications of the same, the concomitant finding was limited, as the effectiveness of the use of humor was not tested. Hence, future studies should employ different methods to investigate any related topic. Second, most of the participants in this study’s sample were volunteer clowns. Future studies must focus on professional clowns in order to evaluate the potential differences between them and the volunteer clowns with respect to the larger study topic. Third, this study was conducted using a sample of Italian participants; further research is essential to confirm these results with regard to those of other cultures and nationalities. 

On the other hand, the present study was the first of its kind to investigate the relationships between the abovementioned eight comic styles, the experience of clown doctors, and the settings of and the audiences addressed by their work. A broad spectrum of professionals in this field, including clowns, trainers, and researchers, stands to benefit from the insights provided by these results. This is an integral contribution of the present study; it claims that individuals entering the realm of clowning in healthcare settings must undergo both psychological and artistic training. Humor is a central element of clowning in healthcare settings; it offers relief to patients and sheds light on their illnesses. Hence, understanding the distinct functions and roles that humor can play in patient interactions is crucial for clown doctors. As clown doctors must interact with different patients during their service, awareness regarding the specific types of employed humor can enhance the effectiveness of their initial and ongoing training. 

## Figures and Tables

**Table 1:** Mean scores and standard deviations (in parentheses) of clown doctors’ experience/characters, group of intervention, and CSM.

	<1–4 Years M (SD)		4–9 Years M (SD)		>9 Years M (SD)		F (5, 210)	η^2^
	W	A	W	A	W	A		
Group intervention								
Children	4.7 (1.03)	4.11 (0.91)	3.8 (1.05)	4.2 (0.70)	4.3 (0.75)	4.3 (0.69)	4.77	0.02
Adolescents	3.0 (0.94)	3.1 (0.97)	3.0 (0.92)	3.5 (1.12)	3.4 (0.85)	3.4 (0.89)	5.16	0.02
Adults	**3.3 ^a^ (1.07)**	**3.2 ^a^ (0** **.98)**	**3.7 ^ab^ (0** **.79)**	**3.7 ^ab^ (0** **.87)**	**3.6 ^b^ (0** **.96)**	**3.6 ^b^ (0** **.82)**	7.21 ***	0.07
Elderly	2.7 (1.26)	3.1 (1.22)	3.3 (1.16)	3.5 (1.09)	3.4 (1.12)	3.2 (0.91)	1.01	0.03
*CSM*								
Fun	5.1 (1.06)	4.8 (1.21)	4.9 (1.08)	5.1 (0.94)	4.9 (1.09)	4.4 (1.08)	2.01	0.02
Humor	5.2 (0.78)	5.3 (0.85)	5.4 (0.70)	5.2 (0.64)	5.3 (0.90)	5.3 (0.74)	0.10	0.00
Nonsense	5.2 (1.05)	5.2 (0.96)	5.3 (1.05)	5.1 (0.91)	5.1 (1.13)	5.2 (0.74)	0.00	0.00
Wit	4.9 (0.83)	4.6 (1.14)	4.8 (0.94)	4.9 (0.95)	5.0 (0.77)	4.6 (0.86)	0.17	0.00
Irony	**4.4 ^ab^ (1.22)**	**4.1 ^ab^ (1.23)**	**4.6 ^b^ (1.09)**	**4.3 ^b^ (1.09)**	**4.1 ^a^ (1.30)**	**3.7 ^a^ (1.03)**	3.39 *	0.03
Satire	4.4 (1.24)	4.3 (1.17)	4.5 (0.99)	4.2 (1.05)	4.0 (1.40)	4.0 (1.02)	2.01	0.02
Sarcasm	**3.4 ^ab^ (1.20)**	**3.4 ^ab^ (1.08)**	**3.8 ^b^ (1.07)**	**3.4 ^b^ (1.01)**	**3.4 ^a^ (1.36)**	**2.7 ^a^ (1.11)**	3.38 *	0.03
Cynicism	**3.7 ^ab^ (1.30)**	**3.5 ^ab^ (1.11)**	**3.9 ^b^ (1.17)**	**3.6 ^b^ (0** **.73)**	**3.5 ^a^ (1.22)**	**3.0 ^a^ (1.06)**	3.12 *	0.03

**Note.** M = Mean; SD = Standard Deviation; W = Whiteface Clown; A = Auguste Clown. *** *p* < 0.001. * *p* < 0.05. Tukey ^a,b^ for experience and characters. Significant differences are noted in **bold**.

**Table 2:** Standardized betas and proportion of variance explained for the regression analyses of levels of experience and group intervention on CSM as predictors (correlation in parentheses).

	Children	Adolescents	Adults	Elderly
CSM				
Experience	0.15 * p = 0.03 (0.114)	0.15 * ***p* = 0.03** (0.149 *) ***p* = 0.03**	0.15 * ***p* = 0.03** (0.151 *) ***p* = 0.03**	0.14 * ***p* = 0.04** (0.141 *) ***p* = 0.04**
*R* ^2^	0.02 * ***p* = 0.03**	0.02 * ***p* = 0.03**	0.02 * ***p* = 0.03**	0.02 * ***p* = 0.03**
Irony	−0.17 ** ***p* = 0.02** (−0.169 *) ***p* = 0.01**	−0.15 * ***p* = 0.03** (−0.145 *) ***p* = 0.04**	−0.01 (−0.005)	0.07 (0.071)
*R* ^2^	0.03 * ***p* = 0.03**	0.02 * ***p* = 0.03**	0.00	0.00

** *p* < 0.01. * *p* < 0.05. Significant differences are noted in **bold**.

## Data Availability

The datasets generated and/or analyzed during the current study are available from the corresponding author upon reasonable request.
